# Fluctuation suppression in microgels by polymer electrolytes

**DOI:** 10.1063/4.0000014

**Published:** 2020-06-15

**Authors:** S. Pasini, S. Maccarrone, N. K. Székely, L. R. Stingaciu, A. P. H. Gelissen, W. Richtering, M. Monkenbusch, O. Holderer

**Affiliations:** 1 Forschungszentrum Jülich GmbH, JCNS at Heinz Maier-Leibnitz Zentrum, Lichtenbergstraße 1, 85747 Garching, Germany; 2 NScD, Oak Ridge National Laboratory, Oak Ridge, Tennessee 37831, USA; 3 Institute of Physical Chemistry, RWTH Aachen University, 52056 Aachen, Germany; 4 Institute of Physical Chemistry, RWTH Aachen University, 52056 Aachen and JARA-SOFT 52056 Aachen, Germany; 5 Jülich Centre for Neutron Science (JCNS) & Institute for Complex Systems (ICS), Forschungszentrum Jülich GmbH, 52428 Jülich, Germany

## Abstract

Structural details of thermoresponsive, cationically poly(N-iso-propylacrylamide-co-methacrylamido propyl trimethyl ammonium chloride) microgels and the influence of the anionic electrolyte polystyrene sulfonate (PSS) on the internal structure and dynamics of the cationic microgels have been studied with a combination of small angle neutron scattering (SANS) and neutron spin echo (NSE) spectroscopy. While SANS can yield information on the overall size of the particles and on the typical correlation length inside the particles, studying the segmental polymer dynamics with NSE gives access to more internal details, which only appear due to their effect on the polymer motion. The segmental dynamics of the microgels studied in this paper is to a large extent suppressed by the PSS additive. Possible scenarios of the influence of the polyanions on the microgel structure and dynamics are discussed.

## I. INTRODUCTION

Microgels are made of chemically cross-linked polymer chains; the typical size of the microgel is of the order of 100–1000 nm.^1,2^ A widely studied class of microgels are thermoresponsive, i.e., the solvent properties change from good to bad solvent when the temperature rises above the volume phase transition temperature (VPTT). Such microgels possess large potential for application in, for example, drug delivery systems, responsive switches, and functional coatings.^3^ Neutron scattering has been used to investigate the structure of microgels with small angle neutron scattering (SANS), where the density profile of microgels with a dense core and a shell with decreasing polymer density has been determined. Also insight into the internal structure of microgels with complex architecture, e.g., core–shell microgels or hollow microgels has been obtained by SANS.^4,5^ Internal density fluctuations and inhomogeneities in microgels fabricated with batch and continuous monomer feeding approaches have been studied recently.^6,7^ The interaction of microgels with electrolytes has been studied with core–shell microgels with a cationic core and an anionic shell, where the uptake and release of polyelectrolytes could be controlled.^8^ Microgels deposited on surfaces as a functional coating have been studied with microscopic techniques^9–11^ and SANS under grazing incidence conditions (GISANS),^12,13^ where deformations of internal heterogeneities can be observed. The dynamics of microgels has been accessed with neutron spin echo (NSE) spectroscopy.^6,7,14–19^ Structural inhomogeneities are often not fully accessible in such systems due to the intrinsic radial averaging of SANS. Still the fluctuations measured at different length scales by NSE have the potential of revealing details of the microgel structure. A rather recent topic is the measurement of polymer chain dynamics at the solid–liquid interface with grazing incidence neutron spin echo spectroscopy (GINSES), the dynamic extension to GISANS. Microgel dynamics at the interface has been studied in Refs. 20 and 21.

An interesting aspect is how the interaction of microgels with the ions can modify the dynamics of the gel. Previous works^18^ have shown that the addition of counterions, like, for example, hexacyanoferrate, can slow down the internal dynamics of PNIPAM-co-MAPTAC-based microgels. This is likely due to the interaction between the ions and the unbalanced charges of the gel that tend to reduce the polymer length in a sort of pinning effect. Thus, the segmental dynamics of the crossed-linked polymer is reduced. Starting from these results, we would like to investigate how polyanions can affect the internal dynamics of PNIPAM-co-MAPTAC in the swollen state. As a method, neutron spin echo provides the best observation window to reveal such dynamical effects.

## II. EXPERIMENTAL

### A. Sample preparation

The microgels used in this study have been synthesized by precipitation polymerization. The amine group of the microgel was quaternized by 80% and is therefore permanently positively charged. Details of the synthesis are described elsewhere^22^ (see also the supplementary material). Polystyrene sulfonate (PSS) with a low molecular weight (3780 g/mol), corresponding to about 20 monomer units, has been used as a polymer electrolyte additive. Deuterated PSS and protonated PSS have been used in order to vary the contrast conditions in the neutron scattering experiments. The cationic poly(N-iso-propylacrylamide-co-methacrylamido propyl trimethyl ammonium chloride) P(NIPAM-co-MAPTAC) microgels used here were synthesized as described in Ref. 22. The microgels were dissolved in 0.1 M NaCl/D_2_O in order to minimize incoherent scattering in the neutron scattering experiments.

### B. Scattering experiments

Small-angle neutron scattering (SANS) experiments were carried out on KWS-2,^23,24^ operated by the Jülich Centre for Neutron Science (JCNS) at the research reactor FRM II of the Heinz Maier-Leibnitz Zentrum (MLZ) in Garching, Germany. To probe a wide *Q*-range (from circa 0.002 up to 0.2 Å^−1^), measurements were performed at sample-to-detector distances of 8 and 30 m on KWS-2 at a wavelength of 5 Å. The following samples were measured at 20 °C, which is below the VPTT of the microgel: PNIPAM-co-MAPTAC amine microgel (0.6% wt) alone and with the addition of either deuterated protonated polystyrene sulfonate (dPSS, hPSS, 0.1% wt). The concentration of the microgel remained the same also after the addition of PSS, for the SANS measurement. As a solvent, D_2_O has been used in order to achieve the best microgel-environment contrast. Salt (NaCl) and HCl were also added. The final pH of the solution was 3. For intensity calibration, empty cell and plexiglass were also measured. Data treatment was performed with QtiKWS10 software.

For the NSE experiments, the measurements have been performed at the SNS-NSE instrument (BL-15) at the spallation neutron source (SNS, Oak Ridge, TN, USA).^25^ Consistent with the SANS experiment, also with NSE, we measured the PNIPAM-co-MAPTAC amine microgel alone and with the addition of either hPSS or dPSS. As the background sample, we measured the solvent D_2_O and NaCl (with HCl, pH = 3) with and without PSS. The mass concentration of the samples was either 1.2% or 0.6% of PNIPAM-co-MAPTAC, the former without PSS, the latter with 0.1% of PSS. Concentrations higher than about 1% wt are desirable for the NSE experiments to have a coherent scattering signal well above the incoherent level. Nonetheless, we had to reduce the concentration of the polymer in the NSE experiment when we added the PSS in order to avoid the formation of a precipitate in the cell. All the measurements have been performed with Hellma 1 mm quartz cells, at a temperature of 20 °C and at different values of the momentum transfer *Q*. The data have been reduced by means of the new program for NSE-data reduction DrSpine.^26^

## III. EXPERIMENTAL RESULTS

### A. Structural analysis

**FIG. 1.**
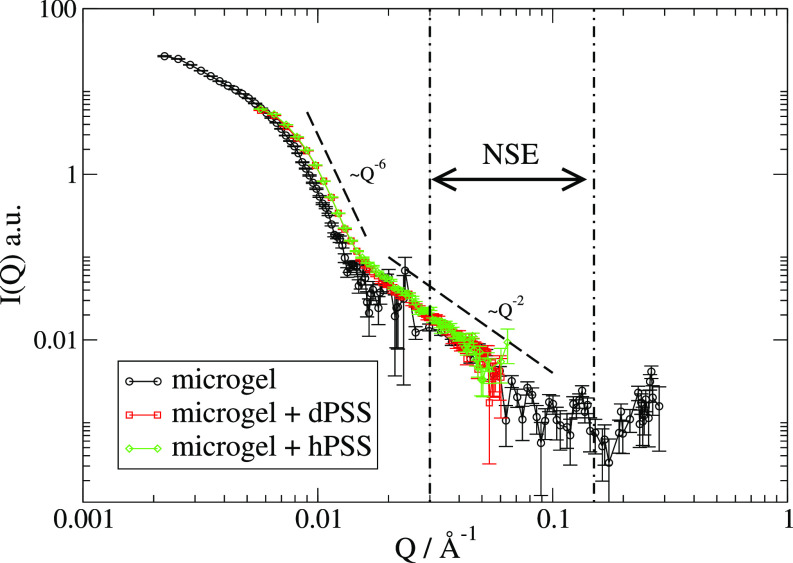
All SANS data for PNIPAM-co-MAPTAC at 20 °C with and without PSS with background subtracted. The region between the dotted-dashed lines marks the *Q* accessible by the NSE technique.

An experiment with small angle neutron scattering is essential to understand whether the addition of the polyanions has caused any structural modification of the microgel. The SANS data for the PNIPAM-co-MAPTAC with and without PSS are shown in Fig. 1. In the region of *Q* of interest for the NSE, the scattered intensity of the PNIPAM-co-MAPTAC presents the expected $Q^{-2}$ power law behavior. For smaller values of the scattering vector, a small elongation of the curve between the $Q^{-2}$ and the $Q^{-6}$ regime can be observed for the samples with PSS.

We use a model that combines the “fuzzy-sphere” model, for the small *Q* regime, with an Ornstein–Zernike term for larger *Q.*^16^ The fuzzy-sphere model

$$I_{S}(Q)=A\int_{0}^{\infty }P{\left(Q,R\right)}^{2}e^{-{\left(\sigma_{\mathrm{surt}}Q\right)}^{2}}G(R,\langle R\rangle ,\sigma_{\mathrm{pol}})dR,$$ (1)

consists of the form factor of a sphere $P(Q,R)=3{\left(\text{sin}\;(QR)-QR\;\text{cos}\;(QR)\right)}^{2}/{\left(QR\right)}^{3}$ convoluted with the Gaussian

$$G(R,\langle R\rangle ,\sigma_{\mathrm{pol}})=\frac{\;\text{exp}\;\left[-{\left(R-\langle R\rangle \right)}^{2}/(2\sigma_{\mathrm{pol}}^{2}\langle R\rangle^{2})\right]}{\sqrt{2\pi \sigma_{\mathrm{pol}}^{2}\langle R\rangle^{2}}},$$ (2)

to account for the polydispersity of the microgel. *σ_surf_* is different from zero for a non-homogeneous cross-linking density. The amplitude *A* is proportional to the number density of the microgel particles, to the volume of the polymer in a particle, and to the contrast between the scattering length of the polymer and the solvent. The Ornstein–Zernike term is given by

$$I_{\mathrm{therm}}=\frac{I_{OZ}}{1+\xi^{2}Q^{2}},$$ (3)

where *ξ* is the correlation length of the thermal fluctuations. The complete scattering intensity is then

$$I(Q)=I_{\mathrm{therm}}(Q)+I_{S}(Q).$$ (4)

Equation (4) is a standard model for PNIPAM-co-MAPTAC in the swollen state.

**FIG. 2.**
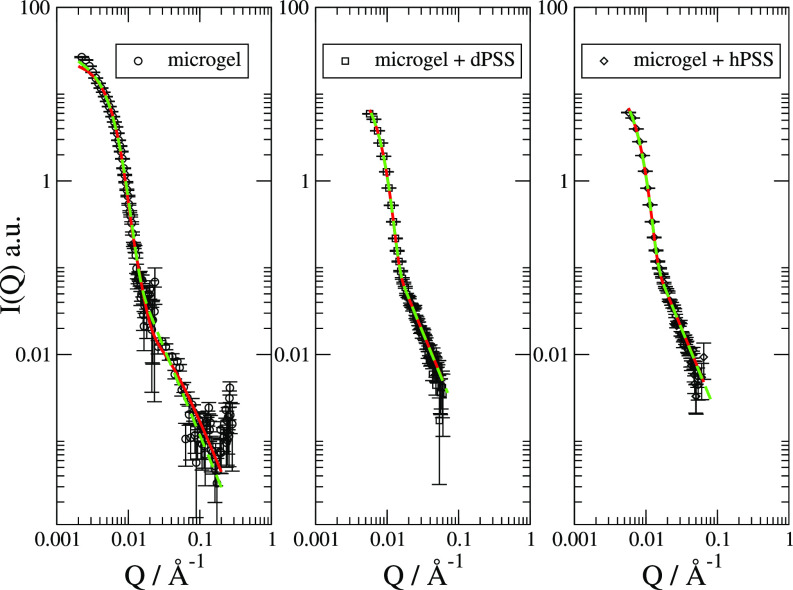
SANS data for the microgel without and with PSS with background subtracted and fitted with Eq. (4) (solid red) compared to the fit where the Ornstein–Zernike function is approximated as in Eq. (5) (dashed green) lines.

Figure 2 shows the SANS data for the microgel with and without the PSS fitted according to 4.

When one applies the Ornstein–Zernike model, one has to keep in mind that *ξ* and *I_OZ_* are correlated if the analysis relies on data with *ξQ* much larger than 1. So the question is whether it is correct to fit them independently. However, when ${\left(\xi Q\right)}^{2}>1$, Eq. (3) can be approximated as

$$I_{\mathrm{therm}}≃\frac{1}{{\left(\Xi Q\right)}^{2}},$$ (5)

where Ξ is defined as $\xi /\sqrt{I_{OZ}}$. The dashed lines in Fig. 2 show that a simple $Q^{-2}$ power law can describe very well the SANS data at larger *Q*. The extracted values for $\frac{1}{\Xi^{2}}$ are reported in Table I.

**TABLE I.** The values for *R_box_*, *R_SANS_* as well as for the rescaled correlation length ($\Xi^{-2}=I_{OZ}/\xi^{2}$) obtained from the model $I(Q)=I_{\mathrm{therm}}(Q)+I_{S}(Q)$ where $I_{\mathrm{therm}}(Q)$ is given by Eq. (5). The digits in brackets for the calculated values of the last column represent the standard uncertainty. The units for Ξ are Å divided by the units of the intensity (cm^–1^).

	*R_box_* (Å)	*R_SANS_* (Å)	$1/\Xi^{2}$	$\xi /\sqrt{I_{OZ}}$
Microgel	385 ± 2	797 ± 4	(11.8 ± 0.1) × 10^−6^	29(1)
Microgel + dPSS	220 ± 10	660 ± 20	(17.8 ± 0.1) × 10^−6^	27(3)
Microgel + hPSS	230 ± 10	680 ± 20	(20.0±0.1) × 10^−6^	22(3)

The density of cross-linking of the microgel is higher in the core and is characterized by a box profile up to a radius given by $R_{\mathrm{box}}=\langle R\rangle -2\sigma_{\mathrm{surf}}$.^16,27^ The parameter *σ_surf_* describes the decreasing of the cross-linking density near the surface of the microgel. At the radius ⟨R⟩, the profile has decreased to half of the core density, while it is already close to zero at $R_{\mathrm{SANS}}=\langle R\rangle +2\sigma_{\mathrm{surf}}$, which is the overall size of the microgel as given by SANS.

### B. Dynamics

For temperatures smaller than the transition temperature VPTT, the microgel is in the so-called swollen state. The main focus of this paper is to study the effect of polyanions on the dynamics of the microgel in this state. For this purpose, we carried out an experiment with the neutron spin echo (NSE), which provides the highest resolution among neutron spectrometers.

**FIG. 3.**
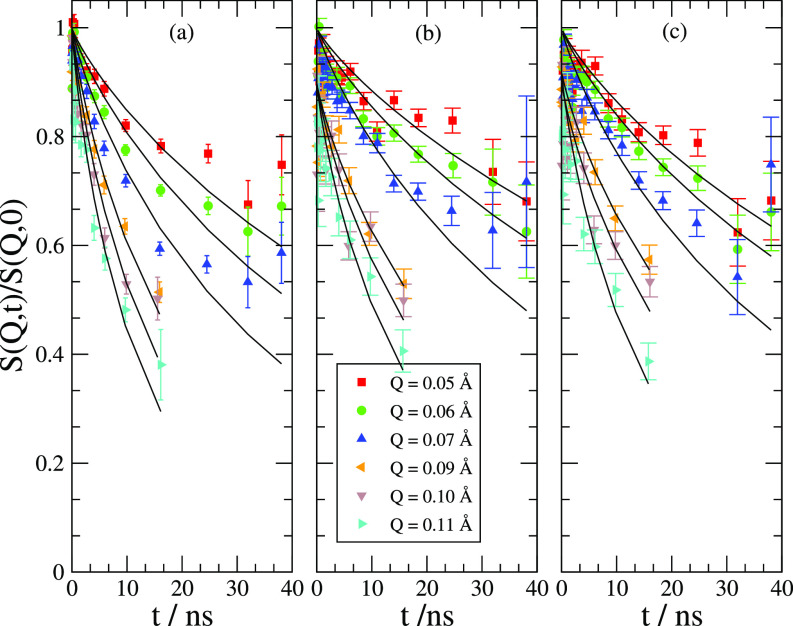
NSE data after background subtraction: (a) PNIPAM-co-MAPTAC, (b) PNIPAM-co-MAPTAC with dPSS, and (c) PNIPAM-co-MAPTAC with hPSS. The fit has been performed with Eq. (6). For the sake of clarity, only some selected curves are shown.

Thanks to the property of the new program for data-reduction DrSpine that allows for a custom binning, up to 20 *Q* bins could be extracted from the experimental data. For the sake of clarity, in Fig. 3 only some selected curves for the normalized intermediate scattering function (S(Q,t)/S(Q,0)) are shown. The curves are already background corrected; as a background, we considered D_2_O with NaCl. In order to control whether contributions from the internal dynamics of the polystyrene sulfonate are present and to what degree these can affect our analysis, a second salt solution containing hPSS (at the same concentration as the one used in the samples with the microgel) was also measured. We could assert that the dynamical content of the PSS at the considered concentration is not relevant and it is indeed comparable with that of the background, i.e., D_2_O with NaCl.

It is widely observed^6,7,14–18^ that NSE data for microgels can be fitted well with a stretched exponential function,

$$\frac{S(Q,t)}{S(Q,0)}=I_{0}\;\text{exp}\;\left[-{\left(\frac{t}{\tau_{0}}\right)}^{\beta }\right],$$ (6)

where typically β=0.85 for Zimm dynamics^28^ and *I*_0_ is the intercept at t→0.

The experimental curves seem to suggest that the PSS tends to slow down the dynamics of the polymer. For a better understanding of the problem, we extract the effective diffusion coefficient from Eq. (6) according to

$$D_{\mathrm{eff}}=\frac{\beta }{\tau_{0}\Gamma (\beta^{-1})Q^{2}}.$$ (7)

**FIG. 4.**
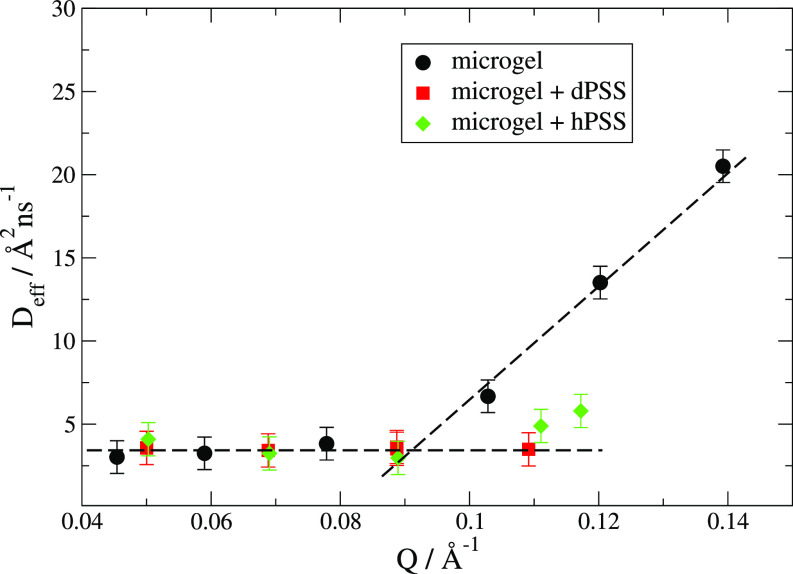
Effective diffusion coefficients as from Eq. (7) with β=0.85.

The extracted values for *D_eff_* are shown in Fig. 4 for the PNIPAM-co-MAPTAC with and without PSS.

The same behavior for *D_eff_* is obtained if the NSE data are fitted with Eq. (6) and *β* = 1.

Equation (6) is used here as a rather generic function for the decay of the intermediate scattering function. It describes the pure segmental chain dynamics with β=0.85 but also a mixture of diffusion and segmental dynamics would result in a slightly modified stretching exponent. Here, we focus on the relaxation time *τ*_0_ as a function of *Q*. The value of *β* does not play a crucial role in the data evaluation and cannot be determined better with the given Fourier time range. The contribution of the diffusion of the overall particle, $S(Q,t)/(S(Q,0)=\text{exp}\;(-DQ^{2}t)$ with the Stokes-Einstein diffusion Coefficient $D=k_{B}T/(6\pi \eta R_{h})$ and the hydrodynamic radius (*R_h_*) of 80 nm from DLS (see the supplementary material), is rather low. This is implicitly included in the *Q*-dependence of the relaxation rate, which is in the beginning dominated not by the overall diffusion, but by the density fluctuations on shorter length scales within the microgel particle, which also have a diffusive character.

## IV. DISCUSSION

The SANS data for all the samples can be described quite well by a combination of fuzzy-sphere and Ornstein–Zernike models. The results of the analysis are a contraction of the average radius suggesting a sort of collapse of the microgel due to the presence of PSS in solution.^29,30^ The SANS data at larger *Q* can be well fitted by a single-parameter model consisting of a $Q^{-2}$ power-law. The values obtained for Ξ reported in Table I provide an estimate of the correlation length if one assumes that the intensity does not change much between the samples. We find that Ξ remains almost unchanged or it rather decreases in the presence of PSS (see), which is consistent with the hypothesis that the microgel shrinks.

The picture delivered by the SANS data is thus a shrunken microgel upon PSS addition. The almost unchanged intensity upon hPSS addition compared to dPSS addition might indicate that it is not uniformly penetrating the whole microgel. We would have expected a higher contrast in the former case if the protonated polyanions fully goes into the full volume of the microgel. A more quantitative SANS analysis could shed light on this question and is left for future investigations.

Internal fluctuations of microgels have been studied with neutron spin echo spectroscopy. The addition of PSS had a strong influence on the local dynamics of the cationic microgel segmental motion and density fluctuations.

The typical transition from diffusive dynamics of density fluctuations to Zimm dynamics of individual segments, which takes place typically at length scales of the microgel mesh size, is suppressed and diffusive fluctuations strongly dominate upon addition of PSS. To our knowledge, this is a new result. The PSS used in this study has a length of approximately 20 monomer units. Polyanions can replace the monovalent counterions inside the microgel, which has been reported, e.g., in Ref. 31 for gels. The loss of monovalent counterions could reduce the osmotic pressure inside the microgel, resulting in a shrinking of it.

The decrease in the correlation length is in agreement with the disappearance of the internal fluctuations in the samples with PSS. Indeed if the complexation of the microgel by the PSS reduces the correlation length, then the Zimm dynamics is to be expected at smaller length scales and therefore, the transition from the *Q*^2^ to the *Q*^3^ behavior in the NSE should occur at larger *Q*.

**FIG. 5.**
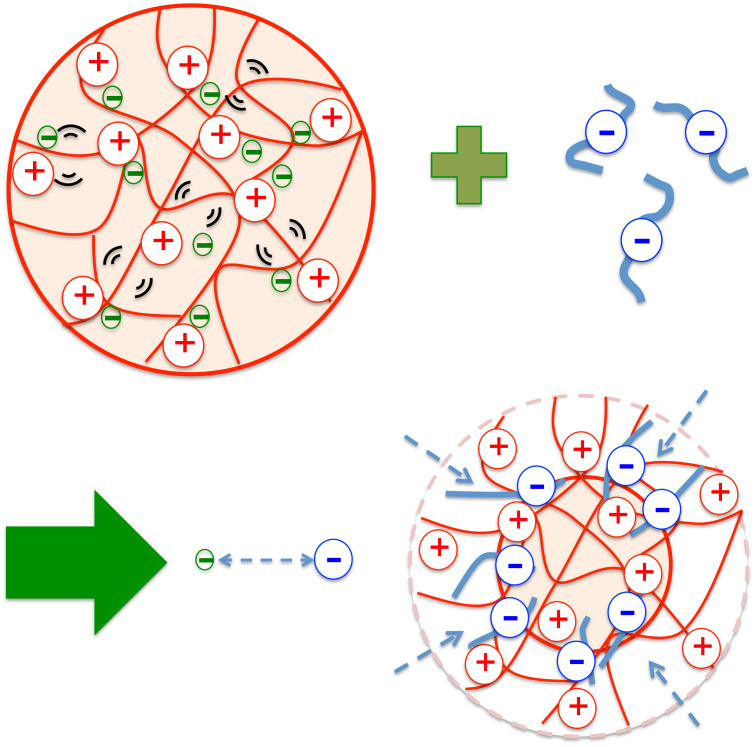
Possible representation of the interaction between the microgel and the PSS: the negative charges in green stand for the monovalent counterions, while the bigger charges in blue represent the PSS polyanions.

The collapse of the microgel by the PSS addition impedes or partially suppresses, some of the internal modes of the polymer (Fig. 5). This way of interacting with counterions appears different with respect to that observed in Ref. 18 between PNIPAM and hexacyanoferrate. The absorbed ions created additional “charge” cross-linkers, acting as an apparent secondary network that slowed down the dynamics of the microgel. But an evident suppression of internal modes was not observed in the case of the relatively small hexacyanoferrate molecules in Ref. 18. The PSS on the other hand seems to suppress Zimm dynamics on the observed length- and time-scales, which might come from the complexation of the polyanions with the cationic microgel.

## V. CONCLUSIONS

In the present study, we investigate the suppression of the internal dynamics of the PNIPAM-co-MAPTAC microgel in the presence of anionic polyelectrolyte polystyrene sulfonate. A combination of small angle neutron scattering and neutron spin echo showed that the microgel, originally in the swollen state, goes through a transition into a sort of collapsed state due to the presence of the PSS. In this state, some of the motions between the cross links are suppressed, at least in the region of *Q* accessible with the NSE technique, and diffusive fluctuations strongly dominate.

## VI. SUPPLEMENTARY MATERIAL

See the supplementary material for details on the synthesis of the microgel and its chemical characterization as well as on the dynamic light scattering (DLS) measurement performed on the sample.

## Data Availability

The data that support the findings of this study are available from the corresponding author upon reasonable request.

## References

[1] R. Pelton , “ Temperature-sensitive aqueous microgels,” Adv. Colloid Interface Sci. 85, 1–33 (2000). 10.1016/S0001-8686(99)00023-8 10696447

[2] A. Pich and W. Richtering , *Chemical Design of Responsive Microgels* ( Springer, 2010), Vol. 234.

[3] M. Karg , A. Pich , T. Hellweg , T. Hoare , L. A. Lyon , J. J. Crassous , D. Suzuki , S. Gumerov , R. A. Schneider , I. I. Potemkin , and W. Richtering , “ Nanogels and microgels: From model colloids to applications, recent developments, and future trends,” Langmuir 35, 6231–6255 (2019). 10.1021/acs.langmuir.8b04304 30998365

[4] J. Dubbert , T. Honold , J. S. Pedersen , A. Radulescu , M. Drechsler , M. Karg , and W. Richtering , “ How hollow are thermoresponsive hollow nanogels?,” Macromolecules 47, 8700–8708 (2014). 10.1021/ma502056y

[5] A. C. Nickel , A. Scotti , J. E. Houston , T. Ito , J. Crassous , J. S. Pedersen , and W. Richtering , “ Anisotropic hollow microgels that can adapt their size, shape, and softness,” Nano Lett. 19, 8161–8170 (2019). 10.1021/acs.nanolett.9b03507 31613114

[6] T. Kyrey , J. Witte , A. Feoktystov , V. Pipich , B. Wu , S. Pasini , A. Radulescu , M. U. Witt , M. Kruteva , R. von Klitzing *et al.*, “ Inner structure and dynamics of microgels with low and medium crosslinker content prepared via surfactant-free precipitation polymerization and continuous monomer feeding approach,” Soft Matter 15, 6536–6546 (2019). 10.1039/C9SM01161G 31355828

[7] J. Witte , T. Kyrey , J. Lutzki , A. M. Dahl , J. Houston , A. Radulescu , V. Pipich , L. Stingaciu , M. Kühnhammer , M. U. Witt *et al.*, “ A comparison of the network structure and inner dynamics of homogeneously and heterogeneously crosslinked PNIPAM microgels with high crosslinker content,” Soft Matter 15, 1053–1064 (2019). 10.1039/C8SM02141D 30663759

[8] A. P. Gelissen , A. Scotti , S. K. Turnhoff , C. Janssen , A. Radulescu , A. Pich , A. A. Rudov , I. I. Potemkin , and W. Richtering , “ An anionic shell shields a cationic core allowing for uptake and release of polyelectrolytes within core–shell responsive microgels,” Soft Matter 14, 4287–4299 (2018). 10.1039/C8SM00397A 29774926

[9] M. U. Witt , S. Hinrichs , N. Möller , S. Backes , B. Fischer , and R. von Klitzing , “ Distribution of CoFe_2_O_4_ nanoparticles inside PNIPAM-based microgels of different cross-linker distributions,” J. Phys. Chem. B 123, 2405–2413 (2019). 10.1021/acs.jpcb.8b09236 30747535

[10] M. F. Schulte , A. Scotti , A. P. Gelissen , W. Richtering , and A. Mourran , “ Probing the internal heterogeneity of responsive microgels adsorbed to an interface by a sharp SFM tip: Comparing core–shell and hollow microgels,” Langmuir 34, 4150–4158 (2018). 10.1021/acs.langmuir.7b03811 29509428

[11] E. Siemes , O. Nevskyi , D. Sysoiev , S. K. Turnhoff , A. Oppermann , T. Huhn , D. Richtering , and W. Wöll , “ Nanoscopic visualization of cross-linking density in polymer networks with diarylethene photoswitches,” Angew. Chem., Int. Ed. Engl. 57, 12280–12284 (2018). 10.1002/anie.201807741 30070009

[12] T. Kyrey , M. Ganeva , K. Gawlitza , J. Witte , R. von Klitzing , O. Soltwedel , Z. Di , S. Wellert , and O. Holderer , “ Grazing incidence sans and reflectometry combined with simulation of adsorbed microgel particles,” Phys. B: Condens. Matter 551, 172–178 (2018). 10.1016/j.physb.2018.03.049

[13] T. Kyrey , J. Witte , V. Pipich , A. Feoktystov , A. Koutsioubas , E. Vezhlev , H. Frielinghaus , R. von Klitzing , S. Wellert , and O. Holderer , “ Influence of the cross-linker content on adsorbed functionalised microgel coatings,” Polymer 169, 29–35 (2019). 10.1016/j.polymer.2019.02.037

[14] T. Hellweg , K. Kratz , S. Pouget , and W. Eimer , “ Internal dynamics in colloidal PNIPAM microgel particles immobilised in mesoscopic crystals,” Colloids Surf., A 202, 223–232 (2002). 10.1016/S0927-7757(01)01077-9

[15] M. Karg , S. Prévost , A. Brandt , D. Wallacher , R. von Klitzing , and T. Hellweg , “ Poly-NIPAM microgels with different cross-linker densities,” *Intelligent Hydrogels* ( Springer, 2013), pp. 63–76.

[16] S. Maccarrone , C. Scherzinger , O. Holderer , P. Lindner , M. Sharp , W. Richtering , and D. Richter , “ Cononsolvency effects on the structure and dynamics of microgels,” Macromolecules 47, 5982–5988 (2014). 10.1021/ma500954t

[17] S. Maccarrone , A. Ghavami , O. Holderer , C. Scherzinger , P. Lindner , W. Richtering , D. Richter , and R. G. Winkler , “ Dynamic structure factor of core-shell microgels: A neutron scattering and mesoscale hydrodynamic simulation study,” Macromolecules 49, 3608–3618 (2016). 10.1021/acs.macromol.6b00232

[18] S. Maccarrone , O. Mergel , F. A. Plamper , O. Holderer , and D. Richter , “ Electrostatic effects on the internal dynamics of redox-sensitive microgel systems,” Macromolecules 49, 1911–1917 (2016). 10.1021/acs.macromol.5b02544

[19] C. Scherzinger , O. Holderer , D. Richter , and W. Richtering , “ Polymer dynamics in responsive microgels: Influence of cononsolvency and microgel architecture,” Phys. Chem. Chem. Phys. 14, 2762–2768 (2012). 10.1039/c2cp23328b 22252036

[20] K. Gawlitza , O. Ivanova , A. Radulescu , O. Holderer , R. von Klitzing , and S. Wellert , “ Bulk phase and surface dynamics of PEG microgel particles,” Macromolecules 48, 5807–5815 (2015). 10.1021/acs.macromol.5b00788

[21] S. Wellert , J. Hübner , D. Boyaciyan , O. Ivanova , R. von Klitzing , O. Soltwedel , and O. Holderer , “ A grazing incidence neutron spin echo study of near surface dynamics in p (MEO 2 MA-co-OEGMA) copolymer brushes,” Colloid Polym. Sci. 296, 2005–2014 (2018). 10.1007/s00396-018-4421-9

[22] A. P. Gelissen , A. J. Schmid , F. A. Plamper , D. V. Pergushov , and R. Walter , “ Quaternized microgels as soft templates for polyelectrolyte layer-by-layer assemblies,” Polymer 55, 1991–1999 (2014). 10.1016/j.polymer.2014.02.062

[23] A. Radulescu , N. K. Szekely , and M.-S. Appavou , “ Heinz maier-leibnitz zentrum *et al.* (2015). KWS-2: Small angle scattering diffractometer,” J. Large-Scale Res. Facil. 1, A29 (2015). 10.17815/jlsrf-1-27

[24] A. Radulescu , V. Pipich , H. Frielinghaus , and M.-S. Appavou , “ KWS-2, the high intensity/wide q-range small- angle neutron diffractometer for soft-matter and biology at FRM II,” J. Phys.: Conf. Ser. 351, 012026 (2012). 10.1088/1742-6596/351/1/012026

[25] M. Ohl , M. Monkenbusch , N. Arend , T. Kozielewski , G. Vehres , C. Tiemann , M. Butzek , H. Soltner , U. Giesen , R. Achten , H. Stelzer , B. Lindenau , A. Budwig , H. Kleines , M. Drochner , P. Kaemmerling , M. Wagener , R. Moeller , E. B. Iverson , M. Sharp , and D. Richter , “ The spin-echo spectrometer at the spallation neutron source (SNS),” Nucl. Instrum. Methods Phys. Res., Sect. A 696, 85–99 (2012). 10.1016/j.nima.2012.08.059

[26] P. A. Zolnierczuk , O. Holderer , S. Pasini , T. Kozielewski , L. R. Stingaciu , and M. Monkenbusch , “ Efficient data extraction from neutron time-of-flight spin-echo raw data,” J. Appl. Crystallogr. 52, 1022–1034 (2019). 10.1107/S1600576719010847 31636520 PMC6782076

[27] C. Scherzinger , P. Lindner , M. Keerl , and W. Richtering , “ Cononsolvency of poly(N,N-diethylacrylamide) (PDEAAM) and poly(N-isopropylacrylamide) (PNIPAM) based microgels in water/methanol mixtures: Copolymer vs core-shell microgel,” Macromolecules 43, 6829–6833 (2010). 10.1021/ma100422e

[28] D. Richter , M. Monkenbusch , A. Arbe , and J. Colmenero , “ Neutron spin echo in polymer systems,” Adv. Polym. Sci. 174, 1–221 (2005). 10.1007/b106578

[29] W. Wong and J. E. Richtering , “ Layer-by-layer assembly on stimuli-responsive microgels,” Curr. Opin. Colloid Interface Sci. 13, 403–412 (2008). 10.1016/j.cocis.2008.04.001 18514212

[30] J. Kleinen , W. Klee , and A. Richtering , “ Influence of architecture on the interaction of negatively charged multisensitive poly(N-isopropylacrylamide)-co-methacrylic acid microgels with oppositely charged polyelectrolyte: Absorption vs adsorption,” Langmuir 26, 11258–11265 (2010). 10.1021/la100579b 20377221

[31] V. A. Kabanov , A. B. Zezin , V. B. Rogacheva , and V. A. Prevish , “ Active transport of linear polyions in oppositely charged swollen polyelectrolyte networks,” Makromol. Chem. 190, 2211–2216 (1989). 10.1002/macp.1989.021900921

